# Contemporary Understanding of miRNA-Based Regulation of Secondary Metabolites Biosynthesis in Plants

**DOI:** 10.3389/fpls.2017.00374

**Published:** 2017-03-29

**Authors:** Om P. Gupta, Suhas G. Karkute, Sagar Banerjee, Nand L. Meena, Anil Dahuja

**Affiliations:** ^1^Division of Quality and Basic Sciences, ICAR-Indian Institute of Wheat and Barley ResearchKarnal, India; ^2^Division of Vegetable Improvement, ICAR-Indian Institute of Vegetable ResearchVaranasi, India; ^3^Division of Biochemistry, ICAR-Indian Agricultural Research InstituteNew Delhi, India; ^4^Division of Basic Sciences, ICAR-Indian Institute of Millets ResearchHyderabad, India

**Keywords:** miRNAs, terpenoids, alkaloids, flavonoids, phenolics, glycosides

## Abstract

Plant's secondary metabolites such as flavonoids, terpenoids, and alkaloids *etc*. are known for their role in the defense against various insects-pests of plants and for medicinal benefits in human. Due to the immense biological importance of these phytochemicals, understanding the regulation of their biosynthetic pathway is crucial. In the recent past, advancement in the molecular technologies has enabled us to better understand the proteins, enzymes, genes, etc. involved in the biosynthetic pathway of the secondary metabolites. miRNAs are magical, tiny, non-coding ribonucleotides that function as critical regulators of gene expression in eukaryotes. Despite the accumulated knowledge of the miRNA-mediated regulation of several processes, the involvement of miRNAs in regulating secondary plant product biosynthesis is still poorly understood. Here, we summarize the recent progress made in the area of identification and characterizations of miRNAs involved in regulating the biosynthesis of secondary metabolites in plants and discuss the future perspectives for designing the viable strategies for their targeted manipulation.

## Introduction

Since the age of human civilization, plants are used as a source of nutrition and medicine, which is evidenced by the numerous texts from China and India (Kirtikar and Basu, 1918; Tang and Eisenbrand, 1992). The nutritional and medicinal properties of the plants are due to the presence of numerous metabolites. These metabolites are of two types: primary and secondary. Unlike primary metabolites, secondary metabolites are a huge group of phytochemicals, which are not directly involved in plant's vital processes such as growth, development, and reproduction (Fraenkel, 1959) but they are major components in defense mechanism of plants in order to protect them from any possible harm in the ecological environment (Stamp, 2003) and other interspecies protection (Samuni-Blank et al., 2012). Humans have exploited secondary metabolites in the form of flavoring agents, fragrances, insecticides, dyes, drugs, etc., More than 100,000 phytochemicals have been isolated from different plant sources so far (Mahajan et al., 2011). These secondary metabolites are broadly categorized as terpenoids, alkaloids, phenolics, glycosides, tannins, and saponins (Verpoorte, 1998). These phytochemicals are synthesized in the plants for a specialized need in a specific set of ecological conditions as their biosynthesis are highly energy consuming. This kind of biosynthesis and accumulation behavior of secondary metabolites in plants is the result of tight regulation of their biosynthetic machinery. Metabolic engineering may further pave a way for enhancing biosynthesis of economically important phytochemicals or for producing desired combinations of such chemicals. One of the ways to tinker with biosynthetic pathways is through modulating miRNA levels as miRNAs are the ultimate regulators in plants.

miRNAs are small (21–24 nucleotides), non-coding, riboregulators that regulate gene expression in eukaryotes (Jones-Rhoades et al., 2006). miRNA is transcribed by RNA polymerase II as a precursor RNA known as the primary miRNA (pri-miRNA), which is subsequently processed by DICER-LIKE 1 (DCL1) to release the mature miRNAs. These mature miRNAs are then loaded into the RISC complex to bind mRNAs for cleavage (Jones-Rhoades et al., 2006). miRNAs are well-known molecules for their role in regulating various plants processes under biotic and abiotic stresses (Gupta et al., 2014a,b; Shriram et al., 2016). Recently, various reports suggested their roles in regulating the biosynthesis and accumulation of secondary metabolites in plants (see review Bulgakov and Avramenko, 2015). In the present review, we have updated the knowledge about present understanding on miRNAs based regulation of biosynthesis and accumulation of secondary metabolites in plants.

## Role of miRNAs in flavonoid biosynthesis

**Figure 1:**
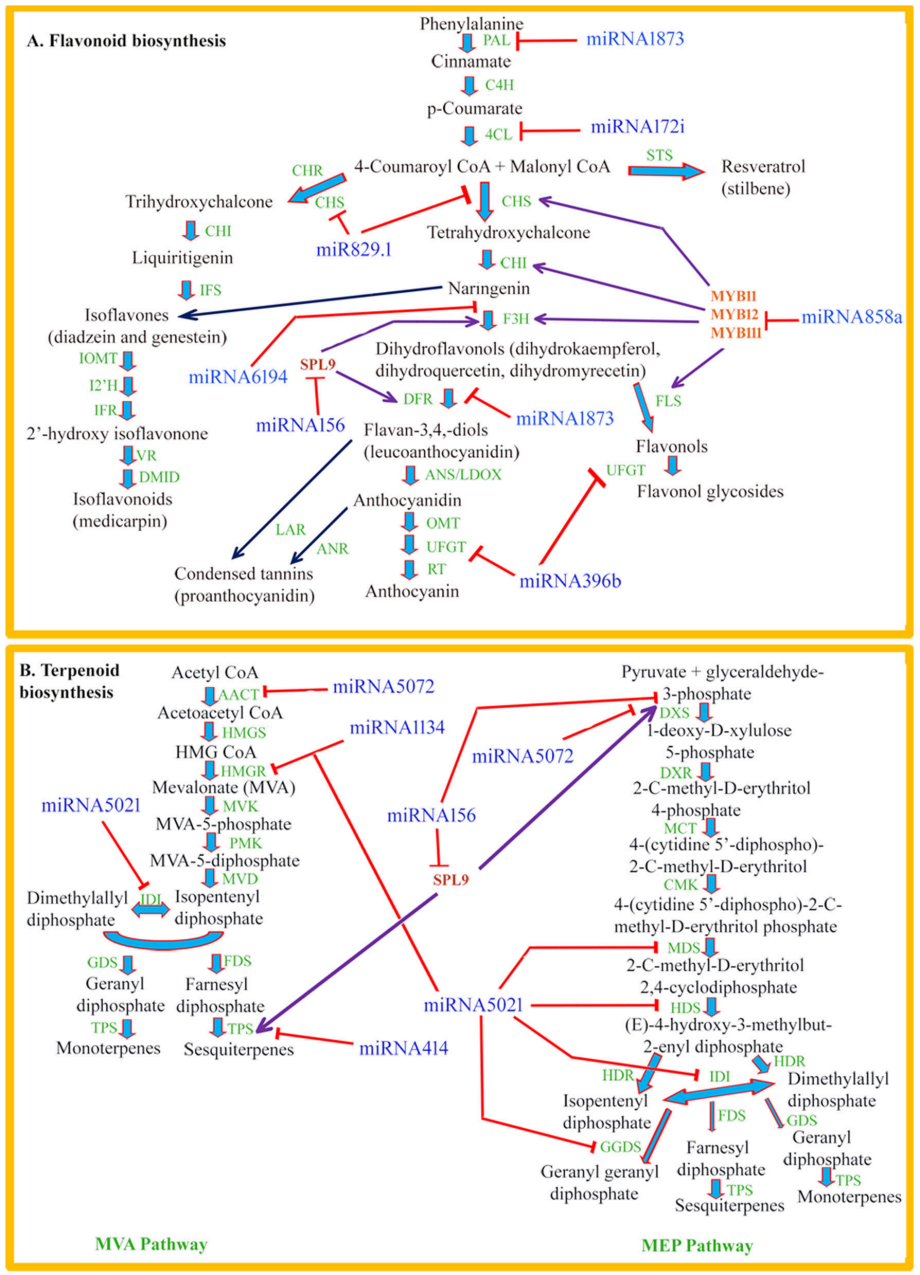
**(A)** Schematic representation of the general phenylpropanoid pathway leading to major branches of flavonoid biosynthesis and their possible interaction with miRNAs. Phe ammonia-lyase (PAL); cinnamate-4-hydroxylase (C4H); 4-coumaroyl:CoA-ligase (4CL); chalcone reductase (CHR), chalcone synthase (CHS); stilbene synthase (STS); chalcone isomerase (CHI); flavanone 3-hydroxylase (F3H); isoflavone synthase (IFS); dihydroflavonol 4-reductase (DFR); isoflavone O-methyltransferase (IOMT); isoflavone 2′-hydroxylase (I2′H); isoflavone reductase (IFR); vestitone reductase (VR); 2′-dihydroxy, 49-methoxyisoflavanol dehydratase (DMID); leucoanthocyanidin dioxygenase (LDOX); O-methyltransferase (OMT); UDPG-flavonoid glucosyl transferase (UFGT); rhamnosyl transferase (RT); flavonol synthase (FLS); leucoanthocyanidin reductase (LAR); anthocyanidin reductase (ANR); anthocyanidin synthase (ANS). **(B)** Schematic representation of biosynthetic pathway of volatile terpenoid and their possible interaction with miRNAs. acetoacetyl-CoA thiolase (AACT); HMG-CoA synthase (HMGS); HMG-CoA reductase (HMGR); mevalonate kinase (MVK); phosphomevalonate kinase (PMK); mevalonate diphosphate decarboxylase (MVD); isopentenyl diphosphate isomerase (IDI); geranyl diphosphate synthase (GDS); farnesyl diphosphate synthase (FDS); terpene synthase (TPS); DOXP synthase (DXS); DOXP reductoisomerase (DXR); 2-C-methyl-D-erythritol 4-phosphate cytidylyltransferase (MCT); CDP-ME kinase (CMK); 2-C-methyl-D-erythritol 2,4-cyclodiphosphate synthase (MDS); (E)-4-hydroxy-3-methylbut-2-enyl diphosphate synthase (HDS); (E)-4-hydroxy-3-methylbut-2-enyl diphosphate reductase (HDR); geranyl geranyl diphosphate synthase (GGDS).

Flavonoids such as flavonols, flavones, isoflavones, anthocyanins, proanthocyanidins, and phlobaphene pigments are low molecular weight phenylpropanoid compounds which are widely distributed throughout the plant kingdom (Taylor and Grotewold, 2005; Lepiniec et al., 2006; Buer et al., 2010). These polyphenolic metabolites play a variety of significant biological roles such as protection against UV radiation, as signaling molecules, as phytoalexins in plant-microbe interaction, and as regulators of phytohormones such as auxin transport in plants (Santelia et al., 2008; Buer et al., 2010). The flavonoid backbone is synthesized by the central phenylpropanoid pathway and different flavonoid metabolites share common enzymes and substrates. Phenylpropanoid pathway is one of the most extensively studied pathways of secondary metabolites for transcriptional regulation in plants (Quattrocchio et al., 2006; Stracke et al., 2007; Li, 2014). In the past few years, scientific endeavors are directed toward understanding the post-transcriptional regulation of this pathway involving miRNAs. The schematic representation of the general phenylpropanoid pathway leading to major branches of flavonoid biosynthesis and their possible interaction with miRNAs has been depicted in Figure 1A.

About 17 SQUAMOSA PROMOTER BINDING PROTEIN-LIKE (SPL) proteins are encoded by the Arabidopsis genome (Riese et al., 2007). These SPL transcription factors are reported to affect numerous processes of plant growth and development, such as vegetative phase transition by enhancing the expression of miRNA172, flowering induction by LEAFY and MADS box genes, embryonic development, cell size, trichome formation, and fertility (Wu et al., 2009; Yamaguchi et al., 2009; Xing et al., 2010; Yu et al., 2010). In addition, miR156 targeted SPL9 protein has been shown to regulate the metabolic flux during flavonoid biosynthetic pathway. Anthocyanins accumulate in an acropetal manner in Arabidopsis stems, with the highest level at the junction between the stem and the rosette leaves. This array of anthocyanin accumulation is regulated by the miR156 targeted SPL9 gene in Arabidopsis (Gou et al., 2011). The tissues having high anthocyanin concentration accumulate higher levels of miRNA156 leading to reduced SPL activity which in turn enhance the expression of F3′H, DFR, and other anthocyanin biosynthetic genes. As a result, dihydroflavonols are directed into the anthocyanin branch. On the other hand, expression of SPLs gradually increases along the growing stem because miR156 levels decline as the plant progresses during development (Gou et al., 2011). Therefore, increased accumulation of SPL leads to decreased expression of anthocyanin biosynthetic genes resulting in the increased production of flavonols by FLS. It has been demonstrated that MYB-bHLH-WD40 transcriptional activation complex is destabilized by SPL9, a target of miRNA156, by competing with bHLHs for their binding to PAP1 which in turn inhibits expression of anthocyanin biosynthetic genes (anthocyanidin synthase, flavanone 3-hydroxylase, dihydroflavonol reductase, and UDP-glucosyl transferase 75C1 etc.) influencing anthocyanin accumulation in *Arabidopsis* (Gou et al., 2011). Similarly, miRNA156-SPL9 pair influences anthocyanin production by targeting dihydroflavonol 4-reductase (Cui et al., 2014). Therefore, an antagonistic relationship exists between anthocyanin and flavonol biosynthesis in Arabidopsis. Recently, Biswas et al. (2016) have computationally identified several miRNAs such as miR172i, miR829.1, miR1438, miR1873, and miR5532 targeting mRNAs coding for enzymes of phenylpropanoid pathway, such as 4-coumarate–CoA ligase, Chalcone synthase, Caffeoyl-CoA O-methyl transferase, Dihydroflavonol 4-reductase C, 2-hydroxyisoflavanone dehydratase respectively in *Podophyllum hexandrum* (Table 1). Overexpression of miR8154 and miR5298b in sub-cultured Taxus cell lines revealed their crucial role in the regulation of taxol, phenylpropanoid, and flavonoid biosynthesis pathways (Zhang et al., 2015). Similarly, several other miRNAs of phenylpropanoid pathway, such as miR395p-3p/ targeting bHLH mRNA in *D. kaki* (Luo et al., 2015), miR396b and miR828a targeting mRNAs coding for Kaempferol 3-O-beta-D-galactosyltransferase and anthocyanin regulatory C1 protein respectively in *R. serpentina* (Prakash et al., 2016), miR858a targeting R2R3-MYB mRNA in *A. thaliana* (Sharma et al., 2016), miR6194 targeting Flavanone 3b-hydroxylase mRNA (F3H) in *H. caspica* (Yang et al., 2015), miR1061-3p and miR1318 in pear fruit (Wu et al., 2014) etc., (Table 1) have been reported.

**Table 1:** **List of miRNAs involved in regulating biosynthesis and accumulation of common secondary metabolites in plants**.

**Sr. no**.	**miRNA**	**Plant species**	**Target**	**Target function**	**Phytochemical biosynthesis**	**Validation/detection**	**References**
**FLAVONOIDS**							
1.	miR156^*^	*A. thaliana*	SPL9	Destabilizes MYB-bHLH-WD40 transcriptional activation complex	Anthocyanin biosynthesis	Transgenic approach	Gou et al., 2011
2.	miR172i	*P. hexandrum*	4-coumarate–CoA ligase	Catalyses the activation of 4-coumarate and other 4-hydroxycinnamates to the respective thiol esters	Flavonoid biosynthesis	Computational	Biswas et al., 2016
3.	miR395p-3p/^*^	*D. kaki*	bHLH	Regulates genes of proanthocyanidin biosynthetic pathway	Proanthocyanidin biosynthesis	Illumina	Luo et al., 2015
4.	miR396b	*R. serpentina*	Kaempferol 3-O-beta-D-galactosyltransferase	Transferase activity, transferring hexosyl groups	Flavonol glycoside	Computational	Prakash et al., 2016
5.	miR828a	*R. serpentina*	Anthocyanin regulatory C1 protein	DNA/chromatin binding	Anthocyanin biosynthesis	Computational	Prakash et al., 2016
6.	miR829.1	*P. hexandrum*	Chalcone synthase	Catalyses the conversion of 4-coumaroyl-CoA and malonyl-CoA to naringenin chalcone	Flavonoid biosynthesis	Computational	Biswas et al., 2016
7.	miR858a^*^	*A. thaliana*	R2R3-MYB transcription factors	Regulate genes of flavonoid biosynthetic pathway	Flavonoid biosynthesis	Transgenic approach	Sharma et al., 2016
8.	miR858b^*^	*D. kaki*	MYB protein	Regulates genes of proanthocyanidin biosynthetic pathway	Proanthocyanidin biosynthesis pathway	Illumina	Luo et al., 2015
9.	miR1438	*P. hexandrum*	Caffeoyl-CoA O-methyl transferase	Cat- alyzes methylation of caffeoyl-CoA to produce feruloyl-CoA.	Lignin biosynthesis	Computational	Biswas et al., 2016
10.	miR1873	*P. hexandrum*	Dihydroflavonol 4-reductase C	Flavanone 4-reductase activity	Flavanoid biosynthesis	Computational	Biswas et al., 2016
		*Z. officinale*	Phenylalanine ammonia lyase (PAL)	Conversion of L-phenylalanine to ammonia and trans-cinnamic acid	Gingerol (phenolic) biosynthesis, Flavanoid biosynthesis	Computational	Singh et al., 2016b
11.	miR5532	*P. hexandrum*	2-hydroxyisoflavanone dehydratase	Catalyses conversion of 2,7,4'-trihydroxyisoflavanone into diadzein	Isoflavonoid biosynthesis	Computational	Biswas et al., 2016
12.	miR6194	*H. caspica*	Flavanone 3b-hydroxylase (F3H)	Catalyses the conversion of flavanone into dihydroflavonol	Biosynthesis of flavonols, anthocyanidins and proanthocyanidins	HiSeq deep sequencing	Yang et al., 2015
13.	CHS-siRNA	*G. max*	Chalcone synthase	Catalyses the conversion of 4-coumaroyl-CoA and malonyl-CoA to naringenin chalcone	Flavonoid biosynthesis	Transgenic approach	Cho et al., 2013; Tuteja et al., 2009
14.	miR1061-3p	*Pyrus spp*	Naringenin 3-dioxygenase	Catalyses the 3-beta-hydroxylation of 2S-flavanones to 2R,3R-dihydroflavonols	Flavonoid biosynthesis	Computational	Wu et al., 2014
**TERPENOIDS**							
15.	miR156^*^	*P. cablin*	SPL9	Activate TPS21 gene	Sesquiterpenoid and triterpenoid biosynthesis	Transgenic approach	Yu et al., 2015
		*M. spp*.	1-deoxy-D-xylulose 5-phosphate synthase (DXS)	Catalyses conversion of 1-deoxy-D-xylulose 5-phosphate into pyruvate and D-glyceraldehyde 3-phosphate	Terpenoid biosynthesis	Computational	Singh et al., 2016a
16.	miR396b	*R. serpentina*	Secologanin synthase	Oxidoreductase activity	Secologanin	Computational	Prakash et al., 2016
17.	miR414	*M. spp*.	Terpene synthase 21 (TPS21)	Catalyses reaction for terpene synthesis	Sesquiterpenoid and triterpenoid biosynthesis	Computational	Singh et al., 2016a
18.	miR838	*Z. officinale*	CYP71	Menthofuran synthase activity	Terpenoid metabo lism	Computational	Singh et al., 2016b
19.	miR4995	*P. kurroa*	3-Deoxy-7- phosphoheptulonate synthase (DAHP synthase)	Catalyses bidirectional conversion of phosphoenolpyruvate + D-erythrose 4-phosphate into 3-deoxy-D-arabino-hept-2-ulosonate 7-phosphate and phosphate	Picroside biosynthesis	Illumina	Vashisht et al., 2015
20.	miR1134	*X. strumarium*	3-hydroxy-3-methylglutaryl coenzyme A reductase (HMGR)	Conversion of HMG CoA into mevalonic acid	Terpenoid backbone biosynthesis	Illumina	Fan et al., 2015
21.	miR5021	*P. hexandrum*	Diphosphomevalonate decarboxylase	Conversion of mevalonate diphosphate (MVAPP) into isopentenyl diphosphate (IPP)		Computational	Biswas et al., 2016
		*C. roseus*	Geranylgeranyl diphosphate synthase (GGPS)	Catalyses the synthesis of GGPP from farnesyl diphosphate and isopentenyl diphosphate			Pani and Mahapatra, 2013
		*M. spp*.					Singh et al., 2016a
		*C. roseus*	GCPE protein	Convserion of CDP-ME 2-phosphate and 2-*C*-methyl-D-erythritol 2,4-cyclodiphosphate (ME-cPP) into hydroxymethylbutenyl 4-diphosphate			Pani and Mahapatra, 2013
			Chloroplast terpenoid cyclase	Terpene synthase activity			Pani and Mahapatra, 2013
		*X. strumarium*	3-hydroxy-3-methylglutaryl coenzyme A reductase (HMGR)	Conversion of HMG CoA into mevalonic acid	Illumina		Fan et al., 2015
		*X. strumarium*	Isopentenyl diphosphate (IPP)/dimethylallyl diphosphate (DMAPP) synthase (IDS)	Isomerization of the carbon–carbon double bond of IPP to create the potent electrophile DMAPP			Fan et al., 2015
		*X. strumarium*	Isopenteyl diphosphate isom- erase (IDI)	Conversion of isopentenyl pyrophosphate (IPP) to dimethylallyl pyrophosphate			Fan et al., 2015
		*M. spp*.				Computational	Singh et al., 2016a
22.	miR5072	*S. miltiorrhiza*	Acetyl-CoA C-acetyl transferase	Conversion of acetyl-CoA into acetoacetyl-CoA	Tanshinones (abietane-type norditerpenoid quinones)	Illumina	Xu et al., 2014
23.	miR5183	*X. strumarium*	Gibberellin 3-oxidase	Catalyses the conversion of precursor GAs to their bioactive forms	Diterpenoid	Illumina	Fan et al., 2015
24.	miR5255	*X. strumarium*	Squalene epoxidase	Oxidize squalene to 2,3-oxidosqualene	Triterpenoid	Illumina	Fan et al., 2015
25.	miR5491	*X. strumarium*	Beta-amyrin synthase	Conversion of (3S)-2,3-epoxy-2,3-dihydrosqualene into beta-amyrin	Triterpenoid	Illumina	Fan et al., 2015
26.	miR5538	*P. hexandrum*	Protein-S-isoprenylcysteine O-methyltransferase	Catalyses the post-translational methylation of isoprenylated C-terminal cysteine residues	Terpenoid backbone biosynthesis	Computational	Biswas et al., 2016
27.	miR6435	*X. strumarium*	Germacrene A oxidase	Oxidations of germacrene A to produce germacrene A acid	Sesquiterpenoid	Illumina	Fan et al., 2015
28.	miR6449	*X. strumarium*	Ent-kaurene synthase	Catalyses bidirectional conversion of ent-copalyl diphosphate into ent-kaurene	Diterpenoid	Illumina	Fan et al., 2015
29.	miR7539	*X. strumarium*	1-deoxy-D-xylulose 5-phosphate synthase (DXS)	Catalyses conversion of 1-deoxy-D-xylulose 5-phosphate into pyruvate and D-glyceraldehyde 3-phosphate	Terpenoid backbone	Illumina	Fan et al., 2015
30.	miR7540	*X. strumarium*	R-linalool synthase	Catalyses the bidirectional conversion of geranyl diphosphate into (3R)-linalool	Monoterpenoid	Illumina	Fan et al., 2015
31.	miRstv__7_^*^	*S. rebaudiana*	*UDP-glycosyl transferase76G1 (UGT76G1)*	Stevioside to Rebaudioside-A	Steviol glycoside biosynthesis	Computational	Saifi et al., 2015
			*Kaurenoic acid hydroxylase* (KAH)	Kaurenoic Acid to Steviol	Steviol glycoside biosynthesis	Computational	Saifi et al., 2015
			*Kaurene Oxidase* (KO)	Kaurene to Kaurenoic Acid	Steviol glycoside biosynthesis	Computational	Saifi et al., 2015
**ALKALOIDS**							
32.	miR13	*P. somniferum*	7-O-methyltransferase (7-OMT)	Conversion of S-reticuline to morphinan alkaloids	BIA biosynthesis	Illumina	Boke et al., 2015
33.	miRX13^*^	*N. tabacum*	Putrescine methyltransferase 2 (PMT2)	Converts putrescine into N-methylputrescine	Nicotine biosynthesis	Illumina	Li et al., 2015
34.	miRX17^*^	*N. tabacum*	Quinolinate phosphoribosyl- transferase 1 (QPT1)	Converts quinolinic acid into NAMN	Nicotine biosynthesis	Illumina	Li et al., 2015
35.	miRX20^*^	*N. tabacum*	Cytochrome P450 monooxygenase (CYP82E4)	Converts nicotine into nornicotine	Nicotine biosynthesis	Illumina	Li et al., 2015
36.	miRX27^*^	*N. tabacum*	Quinolinate phosphoribosyl-transferase 2 (QPT2)	Converts quinolinic acid into NAMN	Nicotine biosynthesis	Illumina	Li et al., 2015
37.	miR408	*P. somniferum*	FAD-binding and BBE domain-containing protein, also known as reticuline oxidase- like protein	Conversion of S-reticuline to (S)-scoulerine	BIA biosynthesis	Illumina	Boke et al., 2015
38.	miR2161	*P. somniferum*	4′ -O- methyltransferase 2 (4-OMT)	Conversion of S-norcoclaurine into S-reticuline	BIA biosynthesis	Illumina	Boke et al., 2015
39.	miR5021	*C. roseus*	UDP-glucose iridoid glucosyltransferase	Transferase activity	Indole alkaloids as well as quinoline alkaloids	Computational	Pani and Mahapatra, 2013
**OTHERS**							
40.	miRn24	*N. tabacum*	Branched-chain amino acid transaminase 3 (BCAT3)	Catalyse the synthesis or degradation of the branched-chain amino acids	Glucosinolate biosynthesis	Computational	Gou et al., 2011
41.	miR826	*A. thaliana*	Alkenyl hydroxalkyl Producing 2 (AOP2)	Side chain modification of Met- derived glucosinolates			Liang et al., 2012
42.	miR5090^*^				Transgenic approach		He et al., 2014

* *In the column number 2 indicates that these miRNAs have been validated for their effect on metabolite accumulation in the plants*.

Further, the use of advanced computational tools complementing the experimental methods has accelerated the accumulation of reports on new as well as existing miRNAs implying their regulatory role during flavonoid pathway in plants. Therefore, further work on functional characterization of these tiny miRNAs-target networks using reverse genetic approach would certainly pave a way for understanding post-transcriptional regulatory mechanism of the flavonoid pathway. This information could further be used for metabolic engineering of the entire pathway for human benefits.

## Role of miRNAs in terpenoid biosynthesis

Owing to their numerous biological roles, isoprene (C5), monoterpenes (C10), and sesquiterpenes (C15) establish the biggest class of plant volatile compounds. In plants, these volatile compounds act as defense molecules against biotic stresses, attracts pollinators and seed disseminators, and help improve thermo-tolerance (Dudareva et al., 2006). In addition, they are used as aroma compounds and natural flavor enhancers which have the beneficial impact on human health (Wagner and Elmadfa, 2003). Considering the importance of these compounds, understanding the regulatory schema of their biosynthetic pathway and accumulation stands on priority. These volatile compounds are synthesized from isopentenyl diphosphate (IPP) and dimethylallyl diphosphate (DMAPP), which are derived from two alternate biosynthetic pathways localized in different subcellular compartments. During the past several years, there has been a significant progress in identification and characterization of genes and enzymes involved in the biosynthesis of volatile terpenoids (Figure 1B), determination of their spatiotemporal expression and compartmentalization, and metabolic engineering. However, the regulatory role of miRNAs in their biosynthesis and accumulation is poorly understood, which opens a new window for further investigations.

Terpene synthases (TPSs) Catalyses the conversion of farnesyl diphosphate (FPP) into sesquiterpenes (C15). Transcription factor SPL9, the target of miRNA156, directly binds to and activates promoter of terpene synthases 21 (TPS21) gene and positively regulates its transcription thereby regulating the synthesis of sesquiterpenoid (Yu et al., 2015). Similarly, miR-4995 was predicted to target mRNA of an enzyme 3-deoxy-7-phosphoheptulonate synthase, which is involved in the picroside biosynthetic pathway in a medicinal herb *P. kurroa* (Vashisht et al., 2015). In addition, Saifi et al. (2015), have mined and validated 11 miRNAs which are involved in steviol glycoside biosynthetic pathway (Table 1) in Stevia and established the relationship pattern with the expression levels of their target mRNAs as well as steviol glycoside contents. Using NGS technology, several miRNAs involved in the sesquiterpene biosynthesis pathway have been mapped and validated in *X. strumarium*. For example, mRNAs of the upstream enzymes in the pathways of terpenoid biosynthesis, including 1-deoxy-D-xylulose 5-phosphate synthase (DXS), 3-hydroxy-3-methylglutaryl coenzyme A reductase (HMGR), isopentenyl diphosphate (IPP)/dimethylallyl diphosphate (DMAPP) synthase (IDS), and isopenteyl diphosphate isomerase (IDI) were predicted to be targeted by miR7539, miR5021, and miR1134 (Fan et al., 2015). The complete list of miRNAs and their target genes have been provided in Table 1. Most recently, bioinformatics approaches have been utilized to mine miRNAs involved in terpenoid metabolism in *Mentha spp*. (Singh et al., 2016a), *Ginger* (Singh et al., 2016b), *C. roseus* (Pani and Mahapatra, 2013), and *P. hexandrum* (Biswas et al., 2016; Table 1).

## The role of miRNAs in the regulating biosynthesis of alkaloid and other N-containing metabolites

Alkaloids are nitrogen containing low molecular-weight compounds which are mostly derived from amino acids. They are known to play significant roles in defense against herbivores and pathogens and are being widely used as pharmaceuticals, stimulants, narcotics, and poisons. Unlike other secondary metabolites, this class is highly diverse and heterogenous in nature and around ~12,000 alkaloids have been characterized till date (Ziegler and Facchini, 2008). These compounds are synthesized through diverse metabolic pathways. Recent genome based technological advancement have led us to add to on our current understanding of their biosynthetic pathways and regulation. However, knowledge on the role of miRNAs during alkaloid biosynthesis and accumulation in plant kingdom has just started to proliferate.

Boke and his coworkers in 2014 have extensively worked on regulation of the alkaloid biosynthesis by miRNA in opium poppy. They identified pso-miR13, pso-miR2161, and pso-miR408 as potential miRNAs involved in the alkaloid biosynthetic pathway. Pso-miRNA2161 targets the mRNA of gene encoding S-adenosyl-L-methionine: 30-hydroxy-N-methylcoclaurine 40-O-methyltransferase 2 (4O MT) enzyme which converts S-norcoclaurine into S-reticuline, an intermediate molecule in benzylisoquinoline alkaloids (BIA) biosynthesis. Similarly, pso-miR13 targets mRNA of 7-O-methyltransferase (7O MT) gene, which converts S-reticuline to morphinan alkaloids. pso-miR408 targets mRNA of reticuline oxidase-like protein which converts S-reticuline to (S)-scoulerine in the BIA pathway. Endogenous target mimicry (eTM) of miRNAs disturbs the function of corresponding miRNAs by inhibiting binding of miRNAs with their authentic target genes (Franco-Zorrilla et al., 2007). Therefore, Li and his co-workers in 2015 have demonstrated that nta-eTMX27 inhibits the expression and function of nta-miRX27 which targets mRNA of quinolinate phosphoribosyl transferase 2 (QPT2) genes leading to enhanced nicotine biosynthesis in the topping treated tobacco. The most recent report by Mao et al. (2017) shows the regulatory role of miR156 targeting SPL9 in the biosynthesis of glucosinolates, which are secondary metabolites functioning as defense metabolites against insect herbivores and pathogens. The SPL9 interacts with JA ZIM-domain (JAZ) proteins, including JAZ3 to control jasmonate synthesis. Increased level of jasmonate further promotes the biosynthesis of glucosinolates. In addition, several other workers have reported numerous miRNAs along with their target genes involved in the alkaloid biosynthetic pathway in *P. hexandrum* (Biswas et al., 2016), *R. serpentina* (Prakash et al., 2016), and *C. roseus* (Pani and Mahapatra, 2013) using computational approaches.

## Modulating secondary metabolites vs. primary metabolite biosynthetic pathways through miRNAs

Unlike secondary metabolites, primary metabolites are required by plants at every stage of their growth and development. And also, the precursor molecules for secondary metabolite biosynthesis are channelized from primary metabolites. Regulation of primary metabolite biosynthetic pathways is well explored at transcriptional, post-transcriptional and now at DNA level, but secondary metabolic pathway are limited at the transcriptional level and recently at post-transcriptional level (miRNA). Therefore, till now, most of the work has focussed on the role of miRNAs during primary metabolism of growth and development. Recently, these miRNAs of primary metabolism along with some other miRNAs are being reported for their crucial role during secondary metabolism, for example, the SPL-miRNA156 system (Gou et al., 2011). Similarly, miR-4995 targets 3-deoxy-7-phosphoheptulonate synthase gene involved in the first step of phenylpropanoid pathway for picrosides I biosynthesis (Vashisht et al., 2015). Being the first enzyme of the pathway, this enzyme holds the key to the progress of pathway as its down-regulation can affect the production of cinnamic acid, thereby affecting picrosides I content. Taking into account the regulatory roles of miRNAs, modification in the expression of such miRNAs would be a promising approach to modulate the biosynthesis of secondary metabolites in plants. SPL9 and TCP3 transcription factors play a major role in secondary metabolism regulation (Gou et al., 2011; Li and Zachgo, 2013) and therefore miRNAs targeting these genes would be an ideal candidate for such approach (Bulgakov and Avramenko, 2015). Nevertheless, identifying and understanding the spatial and temporal expression schema of other miRNAs that might regulate the flux movement at the branch point of primary vs. secondary metabolic pathway and/or secondary metabolic pathway alone would help designing better strategies to favor the biosynthesis of economically important secondary metabolites.

## Conclusion and future directions

Owing to the diversity of the biosynthetic pathway of the secondary metabolites and their biological significance in both plants and human, exploring the regulatory schema of the pathway is crucial. Despite the role of miRNAs during different biotic and abiotic stresses and plant developmental processes, their role in regulating the biosynthesis of secondary metabolites had just started accumulating and it further requires intense and focussed work. Studies on identification of miRNAs and their targets at all possible steps of the pathway and characterizing significant miRNAs-target pairs using reverse genetics is one prime area to decipher the functions of miRNAs. Deep sequencing technologies and the modern computational approaches for miRNA predictions has resulted in the accumulation of huge data on miRNAs. Despite the availability of many computational algorithms, miRNA target identification is still a major challenge. Many miRNA targets which have miRNA binding sites with seed mismatches could not be identified due to the inability of computational tools (Doran and Strauss, 2007). Presently, most of the miRNA target predictions, consider mRNA 3′ UTRs and therefore the genes that are regulated by miRNA through binding in the region other than 3′ UTRs could not be identified (Place et al., 2008; Tay et al., 2008). The miRNAs are part of complex regulatory networks where a single miRNA control 1ots of genes. Thus, modulation of single miRNA expression could result in complicated biological consequences (Lee et al., 2014). This complexity makes functional validation by *knock-out* or overexpression of these predicted miRNAs a challenging issue.

Furthermore, understanding the DNA methylation profiles of plant genomes and their interaction with miRNAs and self-regulation of miRNAs would be an interesting future area of research. In addition, Work on the potential of herbal medicine-derived miRNAs in regulating human health or targeting genes associated with diseases are another emerging area. Such studies would help metabolic engineering of the entire biosynthetic pathway for generating novel phytochemicals or for producing desired combinations of such secondary metabolites.

## Author contributions

OG and AD conceived the idea and designed the manuscript, OG, SB, SK, NM collected literature and wrote the manuscript, OG, SK, and AD critically evaluated the manuscript. All authors approved the manuscript.

### Conflict of interest statement

The authors declare that the research was conducted in the absence of any commercial or financial relationships that could be construed as a potential conflict of interest.
